# Identification and comparison of pandemic-to-symptom networks of South Korea and the United States

**DOI:** 10.3389/fpsyt.2023.1161200

**Published:** 2023-06-23

**Authors:** Mijeong Park, Deachul Seo, Ji Geun Kim, Gayeon Lee, Larkin S. McReynolds, Lawrence Amsel, Hyunjung Yang, Young-Hoon Kim, Sanghoon Han, Soo Hyun Park, Juyoen Hur

**Affiliations:** ^1^Department of Psychology, Yonsei University, Seoul, Republic of Korea; ^2^Department of Psychology, Kyungpook National University, Daegu, Republic of Korea; ^3^Division of Child and Adolescent Psychiatry, Department of Psychiatry, Columbia University-New York State Psychiatric Institute, New York, NY, United States; ^4^Department of Epidemiology, Mailman School of Public Health, Columbia University, New York, NY, United States; ^5^University College, Yonsei University, Seoul, Republic of Korea

**Keywords:** COVID-19 pandemic, anxiety, depression, network analysis, cross-country study

## Abstract

**Background:**

The Coronavirus (COVID-19) pandemic resulted in a dramatic increase in the prevalence of anxiety and depression globally. Although the impact on the mental health of young adults was especially strong, its underlying mechanisms remain elusive.

**Materials and methods:**

Using a network approach, the present study investigated the putative pathways between pandemic-related factors and anxiety and depressive symptoms among young adults in South Korea and the U.S. Network analyses were conducted on cross-country data collected during the COVID-19 lockdown period (*n* = 1,036). Our model included depression symptoms (PHQ-9), generalized anxiety symptoms (GAD-7), and COVID-19-related factors (e.g., COVID-19-related traumatic stress, pandemic concerns, access to medical/mental health services).

**Results:**

The overall structure of pandemic-to-symptom networks of South Korea and the U.S. were found to be similar. In both countries, COVID-related stress and negative future anticipation (an anxiety symptom) were identified as bridging nodes between pandemic-related factors and psychological distress. In addition, worry-related symptoms (e.g., excessive worry, uncontrollable worry) were identified as key contributors in maintaining the overall pandemic-to-symptom network in both countries.

**Conclusion:**

The similar network structures and patterns observed in both countries imply that there may exist a stable relationship between the pandemic and internalizing symptoms above and beyond the sociocultural differences. The current findings provide new insights into the common potential pathway between the pandemic and internalizing symptoms in South Korea and in the U.S. and inform policymakers and mental health professionals of potential intervention targets to alleviate internalizing symptoms.

## 1. Introduction

The Coronavirus (COVID-19) pandemic broke out in December 2019 and has spread to more than 200 countries, resulting in approximately 6.4 million casualties worldwide (1, 2). During the pandemic, there has been a sharp increase in the prevalence of anxiety and depression globally (3–5). A recent study reported that cases of moderate-to-severe depression increased by 25.4% and cases of anxiety disorder rose by 19.5% in 59 countries during the pandemic (6). According to a report by the Organization of Economic Co-operation and Development (7), the COVID-19 pandemic had a greater impact on the mental health of young adults compared to other age groups, with the prevalence of anxiety and depression symptoms almost doubling since before the pandemic for this age group. Young adulthood is the prime time for the emergence and recurrence of anxiety and depressive disorders (8, 9). Young adults, who generally have less secure jobs and are more sensitive to social restrictions, may be more vulnerable to psychological distress caused by vast changes in daily life during the pandemic (10–13). In order to mitigate mental health burden of the pandemic and prepare for future social upheavals, it is critical to understand the mechanisms underlying its impact in this population.

The impact of the pandemic on peoples’ mental health is not independent of their sociocultural contexts. While the COVID-19 pandemic has increased the risk of anxiety and depression worldwide, the way it affects mental health of people across different countries may not be the same. A case in point is South Korea and the United States. There are some contextual differences, including pandemic-related government policies, culture, and access to mental health services, which may have affected the way mental health problems have been manifested in the two countries during the pandemic. For instance, the South Korean government has taken a containment strategy focusing on disease prevention (14–17), whereas in the U.S., a mitigation strategy was implemented at the level of government, that focused on reducing severe cases, while the stringency of the strategy differed widely depending on individual states (14, 18). In addition, people of a collectivistic orientation like that of South Korea tend to show stronger risk perception and a higher sense of social responsibility toward their in-groups compared to a more individualistic culture (e.g., U.S.) (19–21), which may have impacted the psychological vulnerability during the early, adjustment stages of the pandemic. Lastly, differences in mental health care systems and accessibility to available mental healthcare in the two countries might also have contributed to different progress of anxiety/depression during the pandemic.

To our knowledge, there exists only one cross-country study that compared the impact of the pandemic on mental health between South Korea and the U.S. Dean et al. (22) investigated how various factors including demographics, public health strategies and psychological factors during the early stages of the pandemic influenced psychological distress in four countries (South Korea, Hong Kong, France, and the U.S.). Despite the differences in culture and public health strategies, they found overall similarities in the relationship between the pandemic-related factors and psychological distress across these countries. Specifically, younger age, greater concern for COVID-19 and loneliness were identified as the common factors that contributed to deteriorated psychological health during the pandemic (22). However, the dependent variable in their study was a single psychological distress score. The present research examined individual symptoms of anxiety and depression in an attempt to delineate specific pathways from pandemic-related factors to psychological distress.

Network analysis is a promising approach to examine the relationship between pandemic-related factors and anxiety/depression symptoms. The network perspective assumes that mental disorders are emergent phenomena that arise from mutual interactions among multiple symptoms, which is an alternative approach to latent variable models that conceptualize mental disorders as underlying variables that cause a range of psychiatric symptoms (23, 24). Firstly, because the network perspective regards mental disorders as a result of interactions between symptoms, it presumes the role of bridging symptoms that connect the two disorders, or symptoms clusters, to explain their comorbidity (24, 25). Moreover, the network approach can also give insights into the specific pathways between external factors and symptom clusters, by including environmental variables within the network (26). For example, studies have used network analysis to identify specific pandemic-related factors (e.g., fear of infection, isolation and loneliness due to the pandemic) that have direct relationship with anxiety and depressive symptoms within a particular culture or society (27–31). Finally, this approach is useful for detecting core symptoms in the overall network as it reveals the relative degree of connectivity between symptoms (23). As such, unveiling the structure of core and bridging internalizing symptoms and their link with COVID-19 factors using the network approach can provide critical insight into how the pandemic impacts the emotional distress of individuals, and could potentially reveal effective intervention targets.

The aim of the present study was to identify and compare the putative pathways between pandemic-related factors and anxiety and depressive symptoms among young adults in South Korea and the U.S. using a network approach.

## 2. Materials and methods

### 2.1. Study design and participants

A cross-sectional online survey was conducted in South Korea and the U.S. The study was implemented in August 2021 in Korea and September 2020 to May 2021 in the U.S. According to WHO reports, the pandemic in the US was at its peak whereas it had not yet reached its peak in South Korea at the time of data collection.^1^ Participants responded to the survey through an online survey platform, Qualtrics in Korea, and REDCap in the U.S. Given our focus on young adulthood, only the data from undergraduate students attending 4-year university (19–29 years old) were considered. The U.S. study was conducted in accordance with the Declaration of Helsinki and was approved by the Institutional Review Board of Columbia University and the New York State Psychiatric Institute. Informed consent was obtained from all participants involved in the study.

In South Korea, of the 676 participants who responded to the survey, 5 participants (average percentage of missing: 12.6%) were excluded due to their incomplete responses, yielding a final sample of 671 Korean subjects. Data inspection revealed that a large portion of the U.S. data was not usable for the purpose of the current study. In the U.S., of the 1,159 participants who participated in the survey, a total of 803 subjects (average percentage of missing: 69.1%) were excluded from analysis due to incomplete responses (*n* = 738) and unknown gender (*n* = 65). Thus, only a subset (33.3%) of the U.S. sample was used for the current study, yielding a final sample of 365 American subjects (66% White, 11% Asian, 2% Black/African American, 3% Native Hawaiian or Pacific Islander, 18% Native American/Other). The present study was approved by the Institutional Review Board of Yonsei University (South Korea) and Columbia University (U.S.) in their respective countries.

The mean age of South Korean participants was 22.3 years (SD = 2.1 years), and the proportion of female participants was 70.7% (*n* = 474). On the other hand, the mean age of the American participants was 21.2 years (SD = 2.2 years), and 80.8% (*n* = 295) were female. Statistical analysis showed significant differences in both age (*t* = 7.347, *p* < 0.01) and gender (*χ*^2^(1) = 12.283, *p* < 0.01) between the two countries. Follow-up analyses confirmed that the main results remained the same with or without adding these as covariates, suggesting that they did not have a significant impact on the overall network models of the two countries.

### 2.2. Measures

Anxiety symptoms were assessed with the Generalized Anxiety Disorder scale [GAD-7; (32)], a 7-item scale using a 4-point frequency scale ranging from 0 (not at all) to 3 (nearly every day). Depressive symptoms were measured with the Patient Health Questionnaire [PHQ-9; (33)], a 9-item scale ranging from 0 (not at all) to 3 (nearly every day). COVID-19-related variables formulated for the study included the following: COVID-19-related traumatic stress (e.g., hypervigilance, intrusions, avoidance, or nightmares due to issues of COVID-19, etc.), COVID-19 concern about safety and security, COVID-19-related xenophobia, access to mental health services, and access to medical services. Demographic variables included age and gender (male or female). More detailed information on our measures can be found in Supplementary Table S1.

### 2.3. Missing data and imputation

Multiple imputation was adopted which is commonly used in network analysis to address the issue of missing data in the present study. A conservative approach was employed, only including data with missing values of 10% or less. The imputation of missing data was conducted using the *mice* package in R (34).

### 2.4. Analytic strategy

Network analysis was used to examine and compare the pandemic to-anxiety/depression symptoms network in South Korea and the U.S. The overall network structure was estimated separately for each country to identify links within the networks independently. Then, centrality and predictability indices, which reflect the relative importance of each symptom in the network, were calculated to quantify the characteristics of each network. Finally, the network models of the two countries were statistically compared. The accuracy of edge estimation and stability of both centrality indices and network comparison test were additionally assessed to check the robustness of the results.

#### 2.4.1. Network estimation

The R program (version 4.1.3) was used for all statistical analyses. All models were visualized as network graphs using the R-package qgraph (35), where ‘nodes’ represent variables, and ‘edges’ represent the pairwise conditional association between nodes (35, 36). The network structure was estimated with the Mixed Graphical Model (MGM) via regularized generalized regression using the R-package *mgm* (37). MGM was used due to its broad applicability in estimating networks because it allows for the inclusion of diverse variable types and relaxes strict assumptions, such as normality, that are required in traditional models like the Gaussian Graphical Model (36–38). To control spurious associations, the least absolute shrinkage and selection operator (LASSO) was used, which shrinks all edge weights and reduces small weights toward zero (39). Also, as suggested in Epskamp and Fried’s study (2018) (40), Extended Bayesian Information Criterion (EBIC) model with its tuning parameter (*γ* = 0.5) was applied to select the best fitting network (41).

#### 2.4.2. Centrality and predictability indices

Centrality and predictability indices were computed to investigate the structural features of the network (36). Specifically, bridge centrality index indicates a node’s overall connectivity with nodes of the other clusters. It is used to identify nodes that connect different clusters within the network (42) such as bridging symptoms explaining the comorbidity pattern between symptom clusters. The bridge strength index was assessed by using the R-package *networktools* (42, 43). Since visual inspection can lead to misinterpretation of the connections between the different clusters when the network is complex (42, 44), this index was used to objectively quantify and detect nodes that are highly connected to other clusters. As per prior work (42), the top 20% score of bridge strength values were selected as predicted bridging nodes. The strength centrality index, one of the most commonly used indices in network analysis, represents the level of connectivity of a given node with the rest of the nodes in the network, indicating the relative importance of a given node within the network. It was calculated as the sum of all edge weight values connected to the specific node. The predictability was additionally computed by using the R-package *mgm* (37). It represents the extent to which nodes are predicted by other nodes in the network, similar to R2 in regression (45). A high predictability value indicates that the given node can be controlled by its neighboring nodes while a low value stands for the need of direct intervention to the target symptom (46).

#### 2.4.3. Accuracy and stability analyses

In order to check the robustness of the results, accuracy and stability analyses were conducted using R-package *bootnet* (47). To assess the accuracy of network estimation, 1,000 non-parametric bootstraps for each node were performed by computing confidence intervals (CIs), and new datasets were created by resampling observations in the data based on 95% CIs. Narrower CIs corresponds with more accurate estimation of the edges (47). Then, to conduct stability analyses, a case-dropping bootstrap was performed to measure the correlation stability (CS)–coefficient, which indicates the maximum drop percentage of cases to retain a correlation with original centrality indices above 0.7 in at least 95% of the sample (47). Epskamp et al. (47) suggests that a CS-coefficient higher than 0.25 is acceptable but that values greater than 0.5 are preferred. Bootstrapped difference tests were additionally performed to evaluate the differences among edge weights (47).

#### 2.4.4. Network comparison test

The Network Comparison Test (NCT) was conducted using the R-package *NetworkComparisonTest* (48). NCT assesses whether there is a statistically significant difference between networks in aspects of network structure (i.e., overall relations between variables), edge strength, and global strength (i.e., overall connectivity) (48). Since several edges were tested simultaneously, alpha values for multiple comparisons were adjusted using the Holm-Bonferroni correction.

## 3. Results

The descriptive statistics for the anxiety/depressive symptom measures as well as the pandemic-related variables are presented in Supplementary Table S2.

### 3.1. Network of South Korea

#### 3.1.1. Network estimation

**Figure 1:**
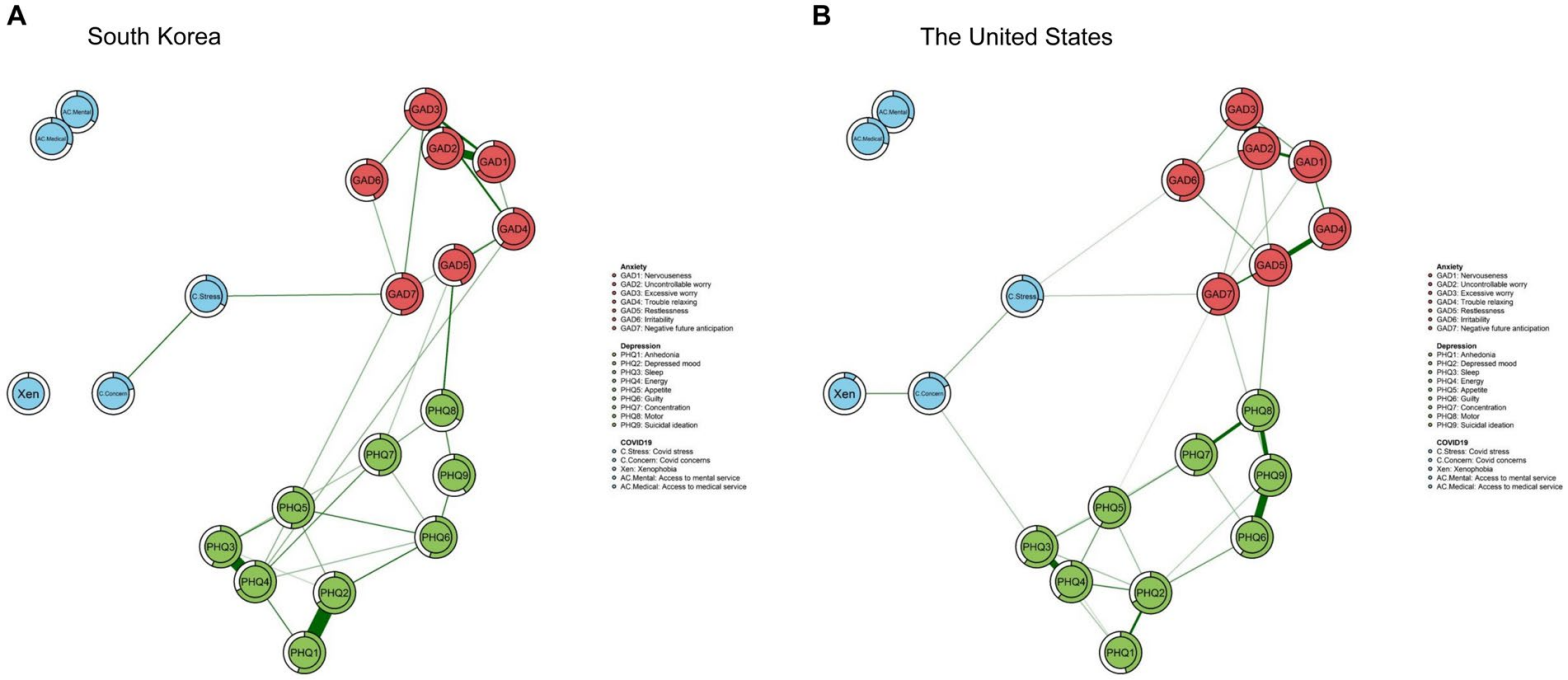
Estimated network of South Korea and the network of the United States. The predictability of each node by other nodes is represented by the rings surrounding each node.

The MGM network for the Korean data is presented in Figure 1A. In this network, anxiety symptoms, depressive symptoms, and pandemic-related factors formed separate clusters with densely interconnected nodes. Within the pandemic-related cluster, the strongest associations were found between ‘Access to mental health service’ and ‘Access to medical service’ (*r* = 0.47). Within anxiety/depressive symptom clusters, two anxiety symptoms, ‘Uncontrollable worry (GAD-2)’ and ‘Excessive worry (GAD-3)’ (*r* = 0.42) showed the strongest association. Across clusters, there were direct edges between ‘COVID-19 stress’ and ‘Irritability (GAD-6)’ (*r* = 0.06) and ‘Negative future anticipation (GAD-7)’ (*r* = 0.08) as well as between ‘COVID-19 concerns’ and ‘Sleep (PHQ-3)’ (*r* = 0.08). Other pandemic factor such as healthcare accessibility did not show any direct relationship with anxiety and depressive symptoms.

#### 3.1.2. Centrality and predictability

**Figure 2:**
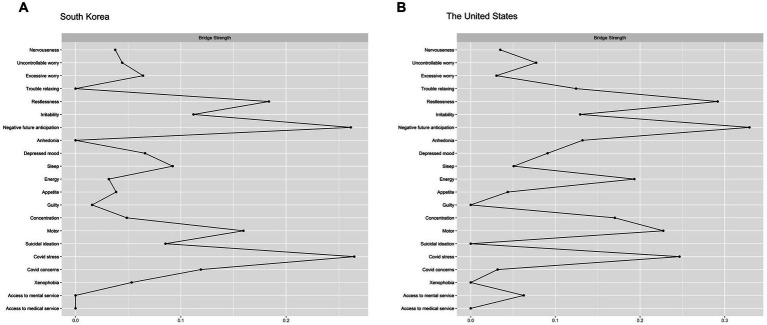
Bridge strength centrality estimates of both networks.

**Figure 3:**
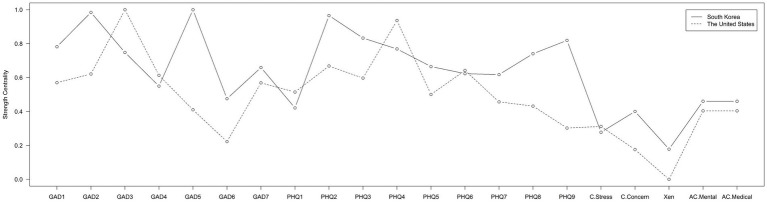
Strength centrality estimates of both networks.

The result of bridge centrality index is shown in Figure 2A. The top bridging nodes which had high bridge centrality values in South Korea were ‘COVID-19 stress,’ ‘Negative future anticipation (GAD-7),’ ‘Restlessness (GAD-5),’ and ‘Motor (PHQ-8)’. As for strength centrality nodes, ‘Restlessness (GAD-5)’ and ‘Uncontrollable worry (GAD-2)’ in the anxiety clusters were identified as the most central nodes in the network of South Korea, followed by ‘Depressed mood (PHQ-2)’ (Figure 3). This indicates that these symptoms are the most influential in maintaining the whole network.

Predictabilities are represented in the rings in the pie chart in Figure 1A. Predictability values ranged from 0.10 to 0.74 (Mean predictability = 0.51 ± 0.18), and the highest predictability value in the overall node was ‘Uncontrollable worry (GAD-2)’ (0.73), which is one of the anxiety symptoms. The lowest predictability index was ‘Xenophobia’ (0.09), which is a pandemic-related factor.

#### 3.1.3. Network accuracy and stability

The results of the accuracy and stability analyses attest to the robustness of the network of South Korea (Supplementary Figures S1, S3, S5). Edge weights showed substantial overlap with the 95% CIs of edge weights, indicating that the edges were stable (Supplementary Figure S1). The bootstrapped difference tests also revealed that most of the comparisons among edge weights were statistically meaningful (Supplementary Figure S3). In addition, case-dropping bootstrap procedure showed that the strength and bridge strength coefficients were 0.75 and 0.36, respectively, which implies that results remained stable after dropping the different proportions of the sample (Supplementary Figure S5).

### 3.2. Network of the United States

#### 3.2.1. Network estimation

The network structure for the U.S. data is presented in Figure 1B. Similar to South Korean data, there was a direct link between the ‘COVID-19 stress’ and ‘Negative future anticipation (GAD-7)’ (*r* = 0.15) but no direct association was found between the pandemic-related factors and depressive symptoms. The strongest association was exhibited in the edge between the two COVID-19-related factors, ‘Access to mental health service’ and ‘Access to medical service’ (*r* = 0.45),’ followed by the two anxiety symptoms ‘Uncontrollable worry (GAD-2)’ and ‘Excessive worry (GAD-3)’ (*r* = 0.39) and the two depressive symptoms ‘Anhedonia (PHQ-1)’ and ‘Depressed mood (PHQ-2)’ (*r* = 0.38).

#### 3.2.2. Centrality and predictability

The bridge centrality result is shown in Figure 2B. Similar to South Korean data, symptoms showing the highest bridge centrality values included ‘Negative future anticipation (GAD-7),’ ‘Restlessness (GAD-5),’ ‘COVID-19 stress,’ and ‘Motor (PHQ-8)’, and indicated the high bridge strength value.

As shown in Figure 3, the highest strength centrality node was ‘Excessive worry (GAD-3)’ in the anxiety symptom cluster, followed by ‘Energy (PHQ-4)’ and ‘Depressed mood (PHQ-2)’ in the depressive symptom cluster. As for the predictability value, the node with the highest predictability score was ‘Excessive worry (GAD-3)’ (0.74), and the node with the lowest predictability was ‘Xenophobia’ (0) (Figure 1B).

#### 3.2.3. Network accuracy and stability

In the network of the U.S., bootstrapped 95% CIs for edges validated the accuracy of the edge-weight estimates (Supplementary Figure S2). The bootstrapped difference test showed that a large proportion of the comparisons among edge weights were significant (Supplementary Figure S4). The CS-coefficient strength centrality and bridge strength was 0.67 and 0.36, respectively, demonstrating the stability of the network model (Supplementary Figure S6).

### 3.3. Estimating the effects of age and gender in network models

In the present study, significant differences were found between the South Korean and American samples in terms of age and gender. Prior to comparing the networks of the two countries, the impact of covariates on the pandemic-to-symptom networks were examined in both countries. Following previous studies (49–51), the pandemic-to-symptom networks of South Korea and the U.S. was re-estimated while controlling for age and gender. Significant correlations were found between the re-estimated network and the original one in both South Korea (*r* = 0.99, *p* < 0.001) (Supplementary Figure S7) and the U.S. (*r* = 0.98, *p* < 0.001) (Supplementary Figure S8). Both statistics and visual inspection of the models confirm that covariates did not have a significant impact on the overall network models in either country.

### 3.4. Network comparison

Finally, the NCT was applied to compare the networks identified in South Korea and the U.S. and revealed no significant differences in network structure (*p* = 0.15) or global strength (*p* = 0.36). This result shows that there is no meaningful difference in overall structure and connectivity between the networks of the two countries.

## 4. Discussion

Using a network approach, the present study investigated the putative pathways between pandemic-related factors and anxiety and depressive symptoms among young adults in South Korea and the U.S. In short, the overall structure of pandemic-to-symptom networks of South Korea and the U.S. were found to be similar. In both countries, anxiety symptoms, depressive symptoms, and pandemic-related factors formed separate clusters. Also, networks from both countries revealed similar common bridging nodes that linked different clusters, and central symptoms were found to be similar in both countries. The implications of the main findings are discussed below.

In both countries, ‘COVID-19 stress’ and ‘Negative future anticipation’ (an anxiety symptom) were identified as bridging nodes and had a direct edge between them, suggesting that this link is likely to function as a key mechanism through which the pandemic affects internalizing symptoms. The COVID-19 stress item measured traumatic stress reactions such as hypervigilance, intrusions, avoidance, or nightmares from issues related to the pandemic over the past month regardless of the presence of direct contact or exposure to the virus. Its strong connection with ‘Negative future anticipation’ suggests that stress induced by the fear of COVID-19 may extend its influence on mental health of young adults primarily by strengthening negative anticipation of the future. Studies have found that, compared to other age groups, young adults tend to experience high levels of pandemic-related stress due to the deprivation of educational/employment opportunities (52, 53). Similarly, with a sample of 18 to 35 years old, Dean et al. (22) found that younger age was a common contributing factor to psychological distress during the early stages of the pandemic across countries with different sociocultural backgrounds. Extending prior work, the present finding suggests that negative future anticipation function as a main trigger for psychological distress experienced by young adults during the pandemic.

It is also noteworthy that ‘Restlessness (GAD-5)’ and ‘Motor (PHQ-8)’ symptoms were identified as bridging symptoms between anxiety and depressive symptom clusters, suggesting that symptoms of physical agitation are strong contributors of the comorbidity between anxiety and depression. The current finding is consistent with prior work demonstrating that physical symptoms show strong bridge centrality indices among anxiety and depressive symptoms (54–56) and that ‘restlessness’ loads on the general distress factor shared in both anxiety and depression (57, 58). Recent studies suggest that physical symptoms observed in comorbid anxiety and depression are reflective of reduced parasympathetic activity for flexible adaptation to stress (59). Though more research is needed, it is possible that the physical symptoms reflect an underlying neurobiological mechanism associated with poor neurovisceral control and emotion regulation generally observed in anxiety and depression. This implicates that physical symptoms may be an important intervention target for comorbid cases of anxiety and depression (56).

Worry-related symptoms were identified as key contributors in maintaining the overall pandemic-to-symptom network in both countries. Specifically, the node ‘Uncontrollable worry (GAD-2)’ showed the highest strength centrality and predictability value in the network of South Korea while the node ‘Excessive worry (GAD-3)’ displayed the highest score in both indices in the network of the U.S. This replicates prior findings that worry-related symptoms were the core symptoms in anxiety-depression network of young adults during the pandemic (60, 61). Since COVID-19 has brought about unprecedented apprehension over future uncertainty, especially for young adults, it is possible that unregulated worrying cascades into a series of internalizing symptoms. Even if worry-related symptoms served as core symptoms in both countries, it is worth noting that the specific elements of worry-related symptoms that served as core symptoms differed between two countries – i.e., ‘excessive worry’ in the U.S. and ‘uncontrollable worry’ in South Korea. According to the initiation-termination (IT) model of worry, which suggests worry as a dynamic process that unfolds over time rather than a static entity (62), “excessive worry” may be more relevant to “proneness of worry initiation when threat is perceived” whereas “uncontrollable worry” is more related to “difficulty in terminating worry.” This suggests that each worry symptom may differ in underlying mechanisms. More research is warranted to investigate this possibility further.

To the best of our knowledge, this is the first comparative network analysis study of the relationship between symptoms of anxiety, depression and factors related to COVID-19 in South Korea and the U.S. The current findings suggest that potential political and societal differences in the two countries (e.g., access to mental/medical health services) was not critically involved in the relationship between the pandemic and internalizing symptoms. Instead, the similar network structures and patterns observed in both countries imply that there may exist a stable relationship between the pandemic and internalizing symptoms above and beyond the sociocultural differences. The current findings suggest that interventions mainly targeting key bridge symptoms (i.e., COVID-19 related stress, negative future anticipation) and worry-related symptoms would most effectively alleviate the impact of the broader internalizing symptom networks during the pandemic in both countries. For instance, treatment elements of cognitive behavioral therapy (CBT) such as exposure-based interventions targeting fear or traumatic symptoms, or cognitive restructuring targeting pathological worry would be helpful to reduce overall comorbidity (63).

The present findings need to be interpreted with the following caveats in mind. Firstly, although Van Borkulo et al. (48) validated NCT results with cases where the sample size of one group was twice as large than the other, the null NCT result should be interpreted cautiously due to the disparity in the number of participants between the two countries. Replication studies are warranted with similar sample sizes in both countries. Secondly, though the null NCT result revealed that there is no statistically meaningful difference in the pandemic-to-symptom networks of the two countries, the difference identified from visual inspection of the networks is worth noting. Under visual inspection, the relationship between COVID-19 concerns and depressive symptoms (i.e., ‘Sleep’) was observed only in South Korea’s network but not in the U.S. data. The disparity between the two results (visual inspection vs. statistical testing) might stem from the methodological difference in network estimation between the Network Comparison Test and Mixed Graphical Model (64). Further studies are needed to address this point since these edges may imply a potential difference between the two networks, even though they were not detected through the statistical comparison test in the current study. Thirdly, in our study, COVID-19 stress was represented as a single score averaging each item for individual pandemic-related trauma symptoms (i.e., proportion of positive symptoms) due to a high number of missing values in the individual items. However, as each item represents slightly different symptoms related to the trauma experience, it will be important for future studies to further investigate the impact of each item separately. Fourthly, the current study did not directly measure contextual factors (e.g., political/cultural factors) that may have influenced the pandemic-to-symptom networks in the two countries. Future studies are warranted to include various contextual factors to gauge their direct impact on the overall networks of the two countries. Lastly, although this study revealed the connection between pandemic-related factors and anxiety and depressive symptoms, it does not confirm any causal relationship since the study was based on a cross-sectional design. Although the results provide evidence of a potentially causal structure, longitudinal studies are called for to investigate whether a clear directionality of influence exists among the anxiety, depressive symptoms, and COVID-19-related factors.

Taken together, the current findings provide new insights into the common potential pathway between the pandemic and internalizing symptoms in South Korea and in the U.S. These observations provide a framework for understanding the impact of economic and/or social upheavals, including but not limited to COVID-19, on mental well-being of young adults and inform policymakers and mental health professionals of potential intervention targets to alleviate internalizing symptoms of this population.

## Data availability statement

The datasets presented in this study can be found at https://osf.io/cs6zm/. Requests to access these datasets should be directed to the corresponding author.

## Ethics statement

The studies involving human participants were reviewed and approved by Institutional Review Board of Yonsei University (South Korea) and Columbia University (U.S.). The patients/participants provided their written informed consent to participate in this study.

## Author contributions

MP and JH designed the study and analytic strategy. JK, HY, GL, LM, LA, and SP collected data. JK cleaned and preprocessed the data. MP performed analyses and created figures and tables. MP and DS drafted the manuscript. JH provided supervision. JH, YK, SH, HY, SP, LM, and LA obtained funding that supported the work. All authors edited the manuscript and approved the final version.

## Funding

This work was supported by the Yonsei Signature Research Cluster Program (Grant No. 2021-22-0005) and the National Research Foundation of Korea (Grants Nos. 2021R1F1A106338512 and 2021S1A5A2A0307022912) in South Korea. The U.S. research was funded by the National Institute on Drug Abuse (NIDA; North Bethesda, MD) grants R01DA038154 (PI: CW Hoven) and R01DA038154-05S2 (PIs: CW Hoven and LM).

## Conflict of interest

The authors declare that the research was conducted in the absence of any commercial or financial relationships that could be construed as a potential conflict of interest.

## Publisher’s note

All claims expressed in this article are solely those of the authors and do not necessarily represent those of their affiliated organizations, or those of the publisher, the editors and the reviewers. Any product that may be evaluated in this article, or claim that may be made by its manufacturer, is not guaranteed or endorsed by the publisher.

## Footnotes

^1^During the data collection period, the pandemic in the US was at its peak with a maximum of 1,667,151 infected cases and 23,212 death cases per week. On the other hand, the trend in the number of confirmed cases and death in South Korea was still increasing (with a maximum of 13,034 infected cases and 64 death cases per week) at the time of data collection.
