# Supplementary material for: Identification and comparison of pandemic-to-symptom networks of South Korea and the United States
Source: Front Psychiatry. 2023 Jun 23;14:1161200. doi: 10.3389/fpsyt.2023.1161200 (PMC10328092; doi:10.3389/fpsyt.2023.1161200)
Supplement: Supplementary file 1 [file Data_Sheet_1.docx]

Supplementary Material

Identification and comparison of pandemic-to-symptom networks of South Korea and the United States

**Mijeong Park^1^, Deachul Seo^1^, Ji Geun Kim^2^, Gayeon Lee^1^, Larkin S. McReynolds^3^, Lawrence Amsel^3^, Hyunjung Yang^4^, Young-Hoon Kim^1^, Sanghoon Han^1^, Soo Hyun Park^1^, and Juyoen Hur^1*^**

*** Correspondence:** Juyoen Hur: [jhur1@yonsei.ac.kr](mailto:jhur1@yonsei.ac.kr)

# Measures

**Table S1.** Details of measures in the MGM model

| Construct | Node name | Node type | Description |
| --- | --- | --- | --- |
| Anxiety | GAD1-GAD7 | continuous | Generalized anxiety was assessed using the Generalized Anxiety Disorder Scale (GAD-7; Spitzer, Kroenke, Williams, & Löwe, 2006), measuring the severity of generalized anxiety disorder over the past two weeks. GAD-7 consists of seven items with a 4-point Likert scale, ranging from 0 (not at all) to 3 (nearly every day). |
| Depression | PHQ1-PHQ9 | continuous | Depression was assessed using the Patient Health Questionnaire-9 (PHQ-9; Kroenke, Spitzer, & Williams, 2001), which is a self-report measure of depression over the previous two weeks. PHQ-9 consists of nine items with a 4-point Likert scale, ranging from 0 (not at all) to 3 (nearly every day). |
| Covid stress | C.Stress | continuous | Covid-19-related traumatic stress was measured using the Primary Care PTSD Screen for DSM-5 (PC-PTSD-5; Prins et al., 2016), which was designed to identify traumatic stress reactions (e.g., nightmares, avoidance, hyperarousal, numbing, and guilt). PC-PTSD-5 consists of five items with yes/no response options. In this study, percentages of yes responses were calculated to assess the severity of pandemic-related traumatic stress. |
| Covid concerns | C.Concern | continuous | Covid-19 concerns items formulated for the study consist of six items: “I am worried about catching the virus”, “I am worried that I can't keep my family safe from the virus”, “I am worried that our healthcare system won't be able to protect my loved ones”, “I am worried that basic hygiene (e.g., handwashing) is not enough to keep me safe from the virus”, and “I am worried that social distancing is not enough to keep me safe from the virus”. Response options were 0 (not at all) to 4 (extremely). In this study, the average score of all items was used to measure the level of covid concern. |
| Xenophobia | Xen | continuous | Xenophobia items were formulated to measure the pandemic-related xenophobic reaction, consisting of six items: “I am worried that foreigners are spreading the virus in South Korea/U.S.”, “If I went to a restaurant that specialized in foreign foods, I'd be worried about catching the virus”, “I am worried about coming into contact with foreigners because they might have the virus”, “If I met a person from a foreign country, I'd be worried that they might have the virus”, “If I was in an elevator with a group of foreigners, I'd be worried that they're infected with the virus”, and “I am worried that foreigners are spreading the virus because they're not as clean as we are”. Response options were 0 (not at all) to 4 (extremely). In this study, the average score of all items was used to measure the level of covid concern. |
| Access to mental service | AC.Mental | continuous | Accessibility to mental health services was measured using the item “I know how to get mental health services”. Responses options were 1 (strongly agree) to 4(strongly disagree). In this study, responses were reverse-coded. |
| Access to medical service | AC.Medical | continuous | Accessibility to medical services was measured using the item “I know how to access a medical provider for physical health needs”. Responses options were 1 (strongly agree) to 4(strongly disagree). In this study, responses were reverse-coded. |

# Results

## Descriptive Statistics

**Table S2**. Descriptive statistics of measurement items

| Node | Label | South Korea | | United States | |
| --- | --- | --- | --- | --- | --- |
|  |  | M | SD | M | SD |
| GAD1 | Nervousness | 0.61 | 0.83 | 1.86 | 1.01 |
| GAD2 | Uncontrollable worry | 0.59 | 0.89 | 1.58 | 1.06 |
| GAD3 | Excessive worry | 0.93 | 0.96 | 1.84 | 1.00 |
| GAD4 | Trouble relaxing | 0.50 | 0.82 | 1.64 | 1.04 |
| GAD5 | Restlessness | 0.29 | 0.65 | 1.17 | 1.11 |
| GAD6 | Irritability | 0.64 | 0.88 | 1.53 | 1.02 |
| GAD7 | Negative future anticipation | 0.35 | 0.72 | 1.35 | 1.12 |
| PHQ1 | Anhedonia | 0.61 | 0.83 | 1.39 | 1.12 |
| PHQ2 | Depressed mood | 0.62 | 0.84 | 1.49 | 1.09 |
| PHQ3 | Sleep | 0.77 | 0.97 | 1.75 | 1.17 |
| PHQ4 | Energy | 0.79 | 0.95 | 1.81 | 1.12 |
| PHQ5 | Appetite | 0.56 | 0.85 | 1.48 | 1.16 |
| PHQ6 | Guilty | 0.41 | 0.74 | 1.47 | 1.17 |
| PHQ7 | Consideration | 0.35 | 0.70 | 1.40 | 1.18 |
| PHQ8 | Motor | 0.22 | 0.60 | 0.65 | 0.98 |
| PHQ9 | Suicidal ideation | 0.25 | 0.62 | 0.59 | 0.92 |
| C.Stress | Covid stress | 0.22 | 0.27 | 0.41 | 0.32 |
| C.Concern | Covid concerns | 1.98 | 1.15 | 1.62 | 1.16 |
| Xen | Xenophobia | 1.70 | 0.99 | 0.27 | 0.55 |
| AC.Mental | Access to mental service | 2.44 | 0.87 | 3.08 | 0.94 |
| AC.Medical | Access to medical service | 2.94 | 0.82 | 3.34 | 0.85 |

PHQ-9: the 9-item Patient Health Questionnaire; GAD-7: 7-item Generalized Anxiety Disorder Scale

## Accuracy of Edge-Weights

We estimated the accuracy of edge weights using the R package ‘bootnet’ (Epskamp, Borsboom, & Fried (2018). Figure S1 and S2 present the results of the 1000 non-parametric bootstraps. In the figure, the red line represents the estimate of the edge stability, and the gray area represents the 95% confidence intervals for the estimates. The value of the edge stability (red line) falls within the bootstrapped confidence intervals (grey area), indicating accurate edge-weight estimations.


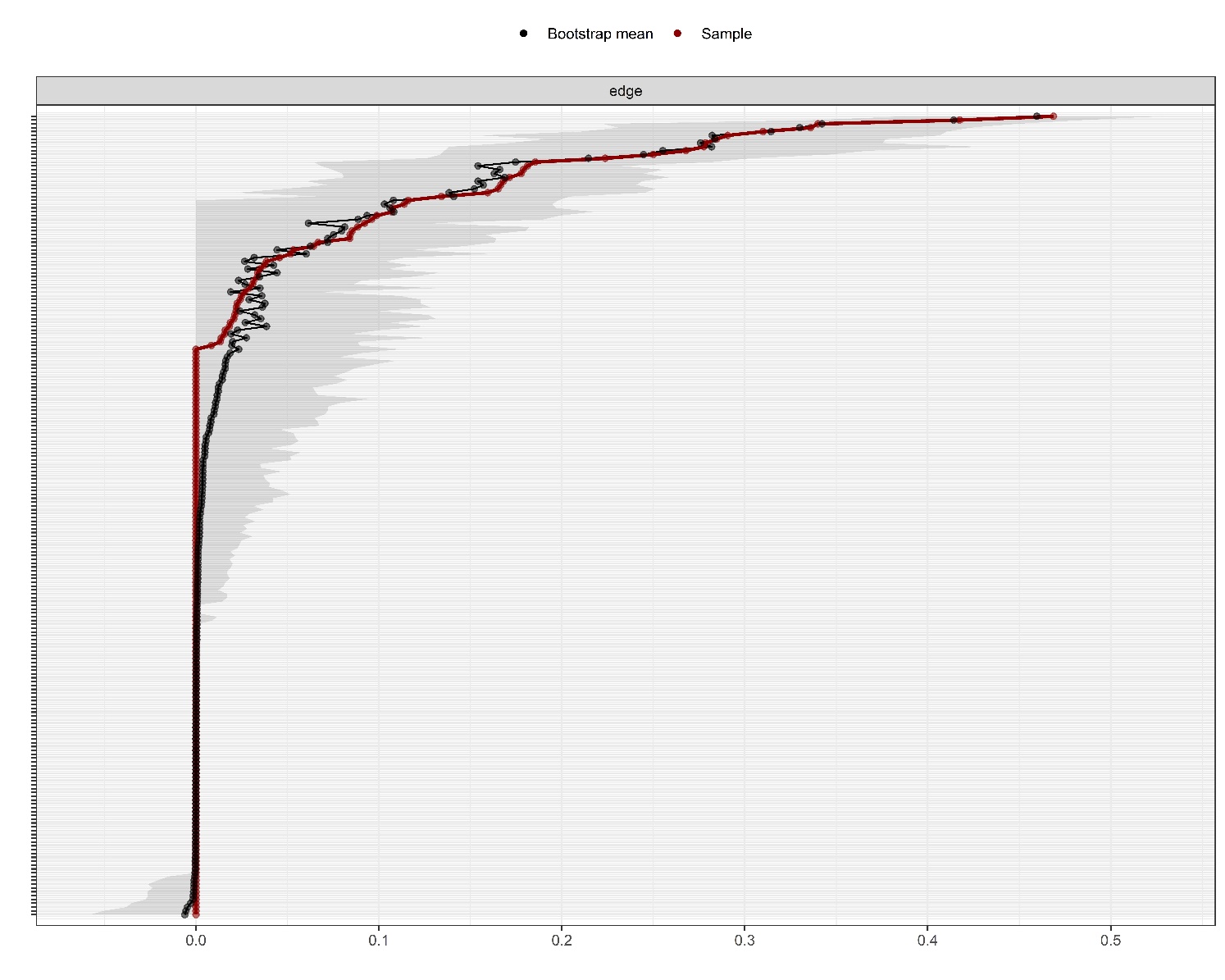


**Fig. S1**. Accuracy of the edge-weight parameters and bootstrapped confidence intervals for the network of South Korea.


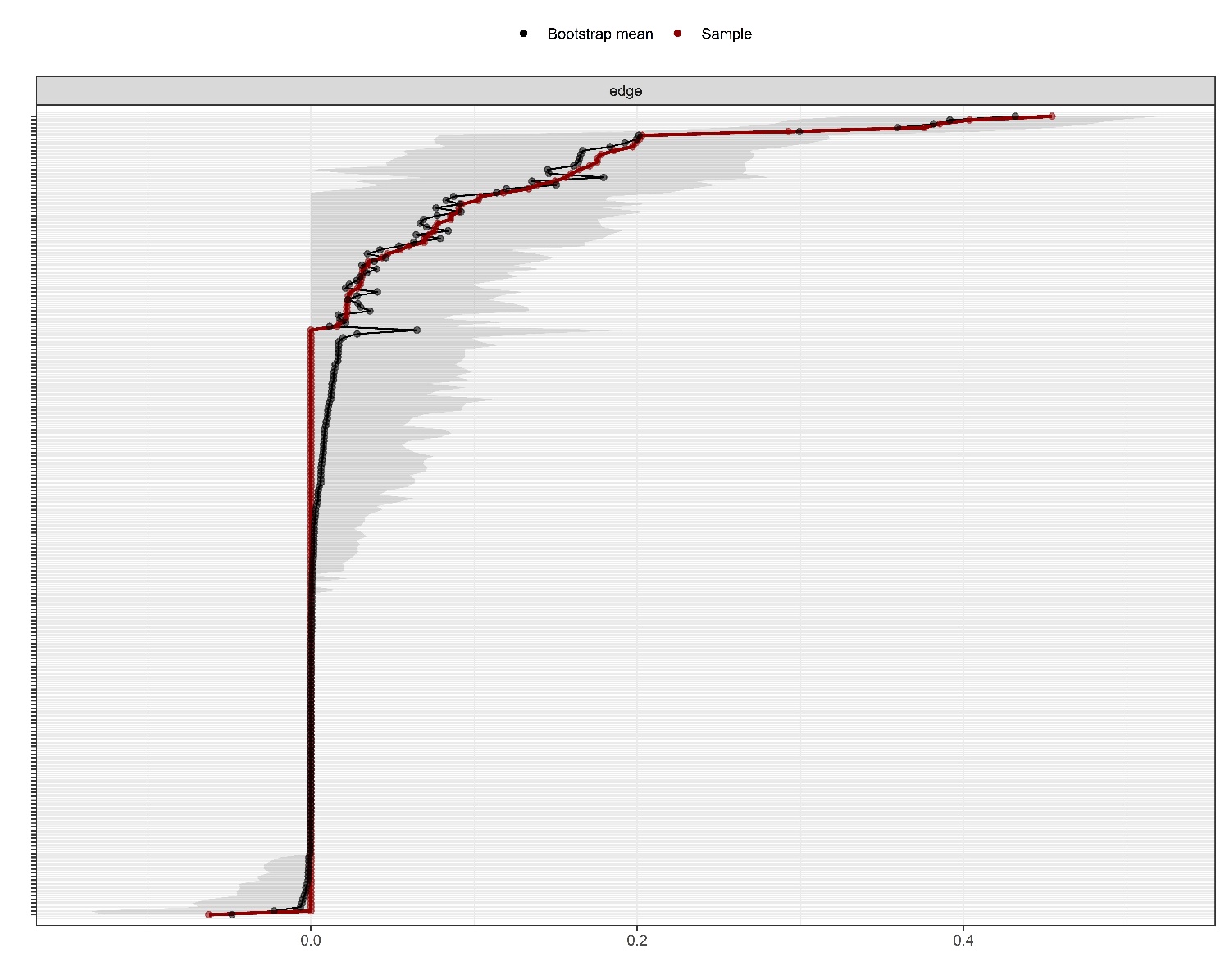


**Fig. S2**. Accuracy of the edge-weight parameters and bootstrapped confidence intervals for the network of the United States.

## Differences between Edge-Weights

We estimated the edge-weight differences using the R package ‘bootnet’ (Epskamp et al., 2018). Gray boxes represent edges that do not significantly different from each other. Black boxes indicate edges that differ significantly from each other. Blue boxes represent positive correlations and red boxes indicate negative connections.


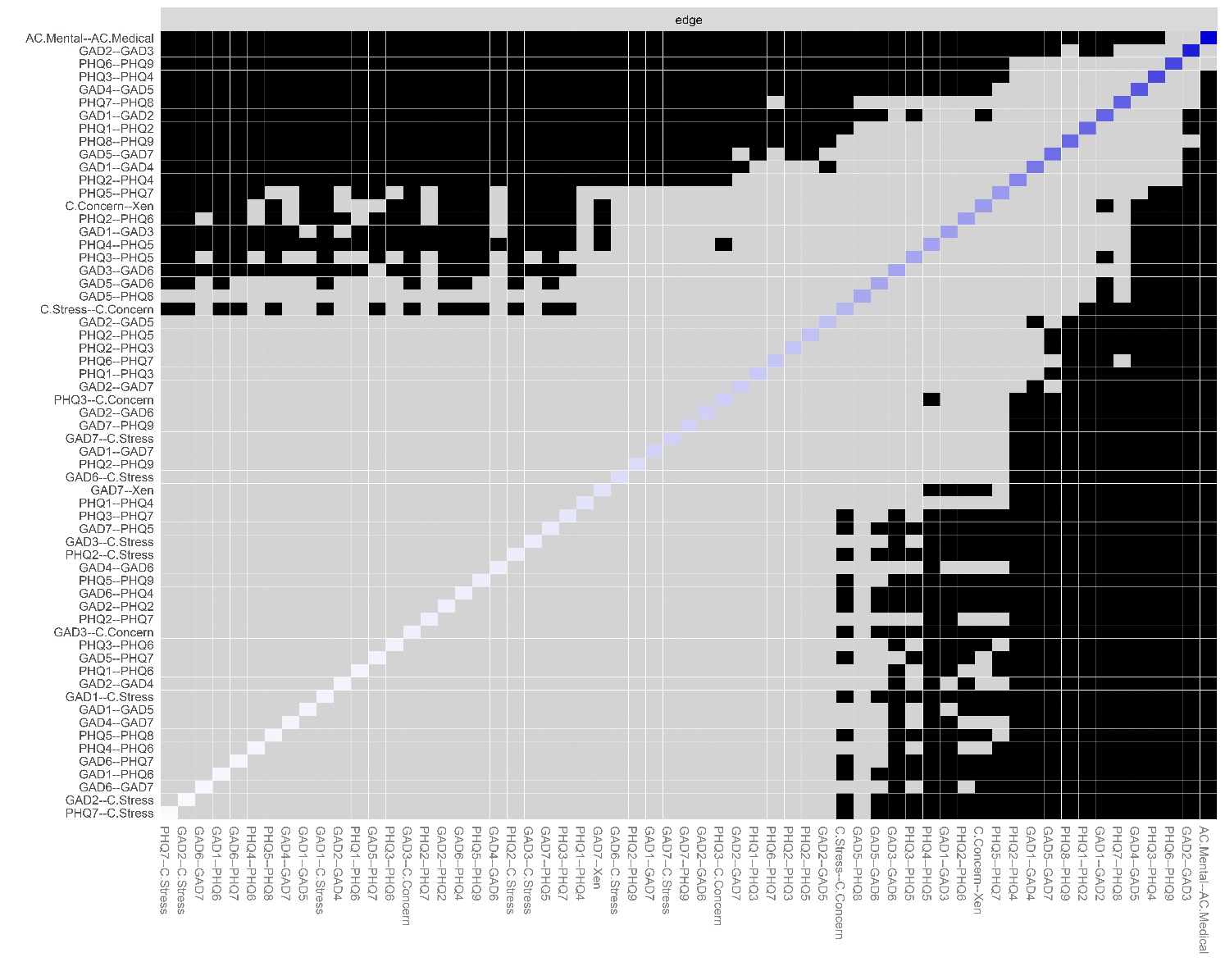


**Fig. S3.** Edge-weight difference test for the network of South Korea.


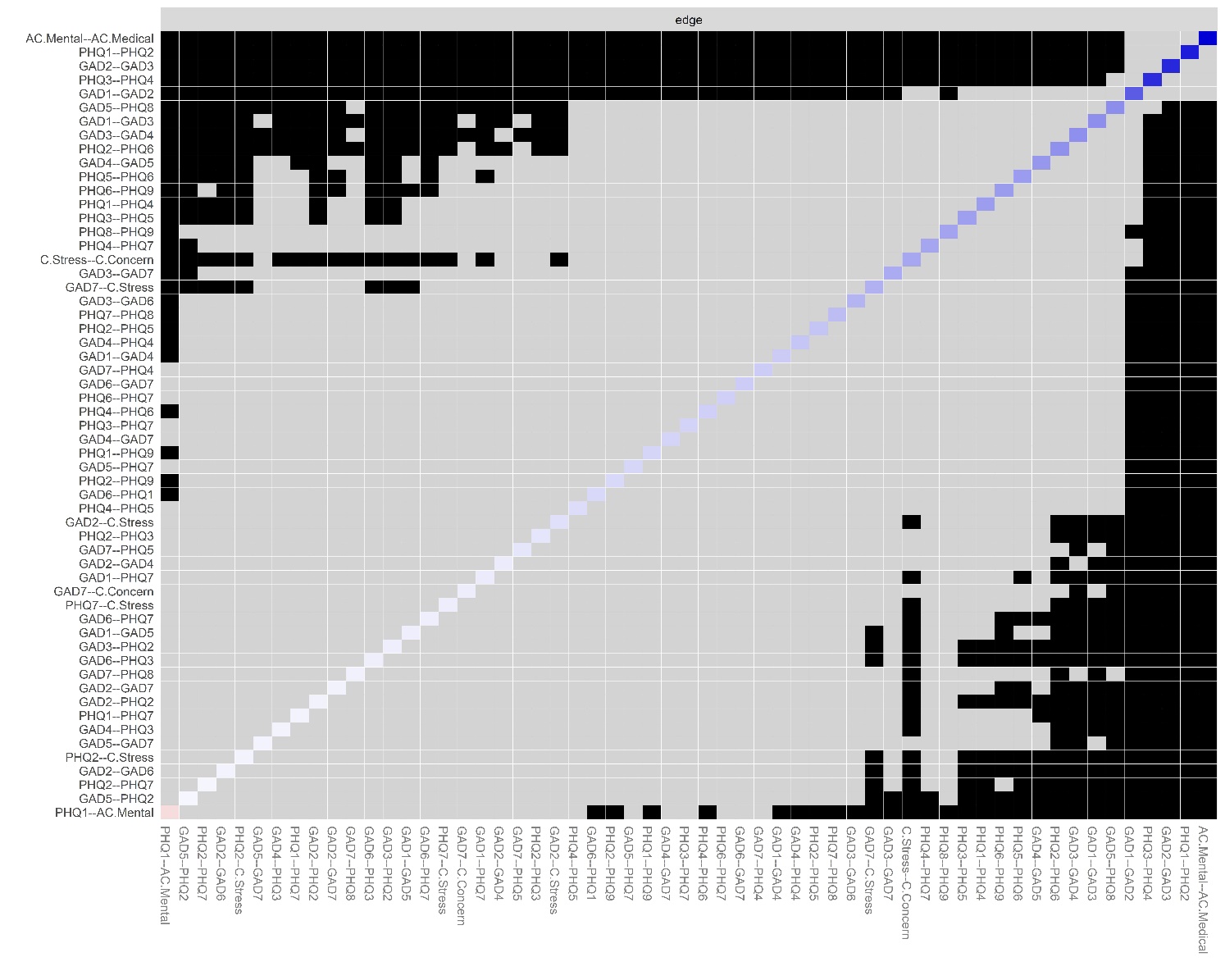


**Fig. S4.** Edge-weight difference test for the network of the United States.

## Stability of Centrality Indices

To assess the stability of centrality indices, the case-dropping subset bootstrap was implemented using the R package ‘bootnet’ (Epskamp et al., 2018). The x-axis indicates the percentage of cases of the original sample and the y-axis represents the correlation between the centrality indices from the original network and from the re-estimated network. In the figure, the blue and red lines represent the correlations of strength and bridge strength respectively, and the blue and red areas represent the 95% confidence intervals for estimates.


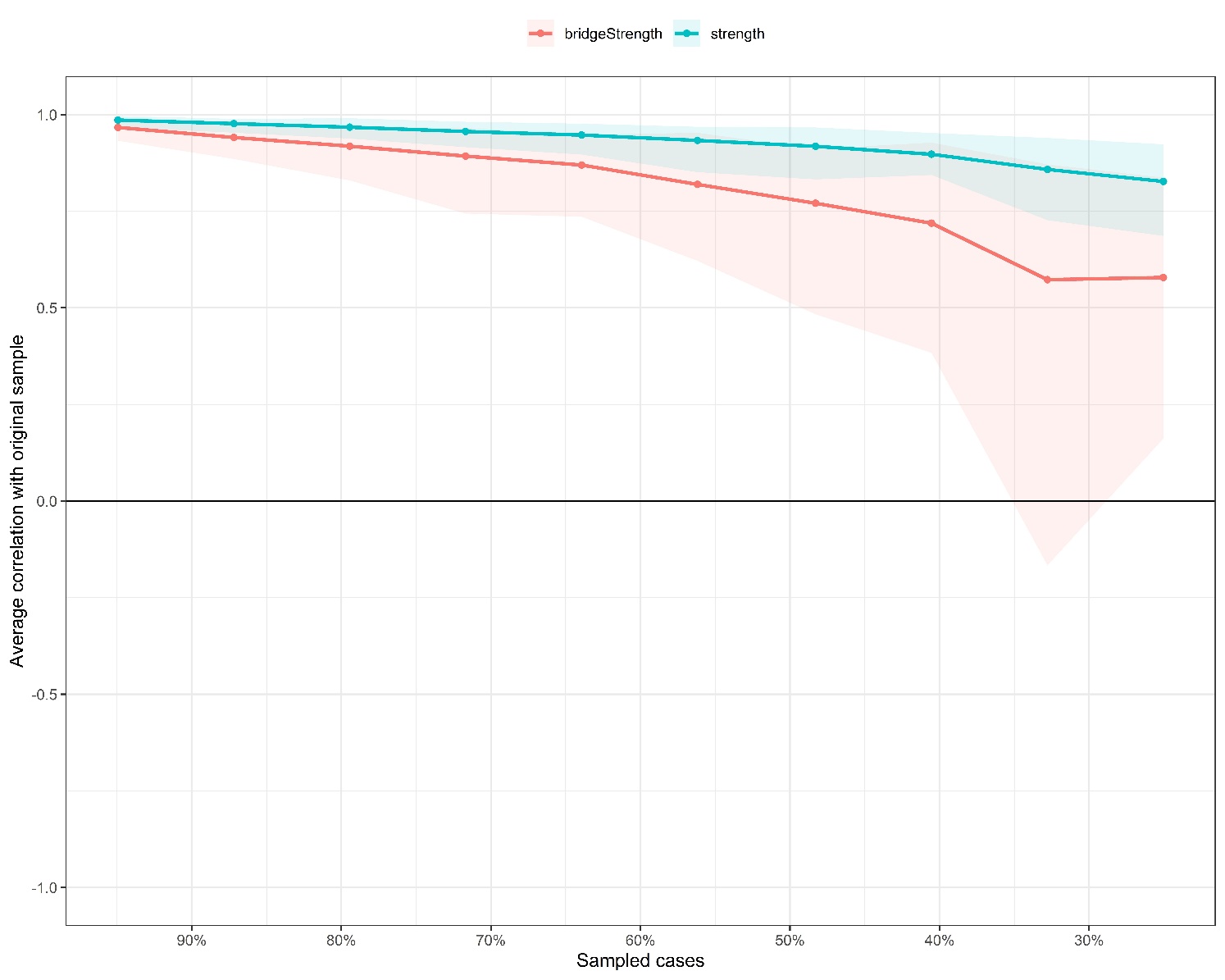


**Fig. S5**. Stability of centrality indices (strength, bridge strength) by case-dropping bootstrapping for the networks of South Korea***.***


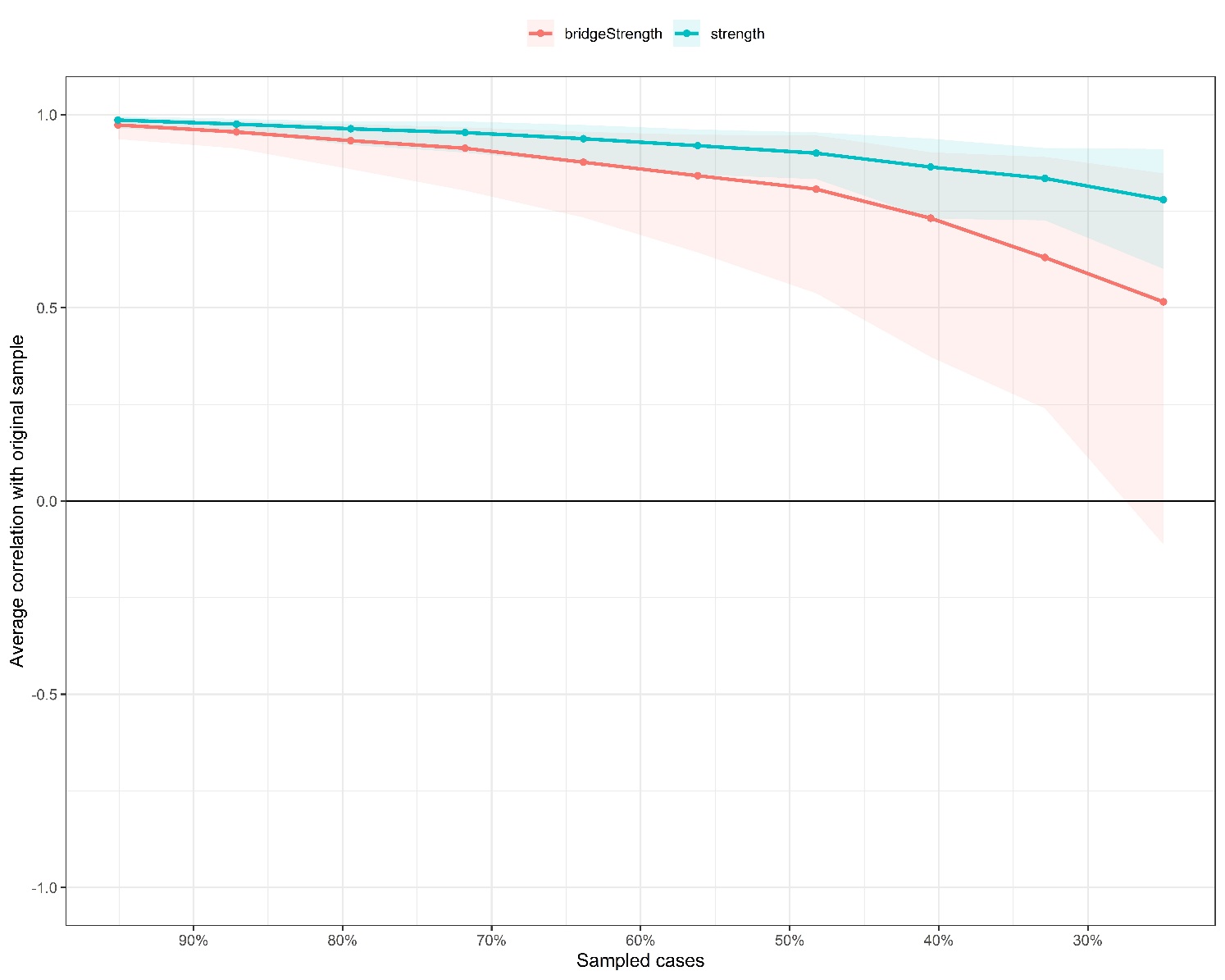


**Fig. S6**. Stability of centrality indices (strength, bridge strength) by case-dropping bootstrapping for the networks of the United States***.***

## Estimating the Effects of Age and Gender in Network Models

Based on Dalege et al (2017), we re-estimated the network while controlling for age and gender. Specifically, we obtained the weighted adjacency matrix of the network with covariates and correlated it with the weighted adjacency matrix of the network without covariates.

The following figures represent the re-estimated networks after controlling for covariates.


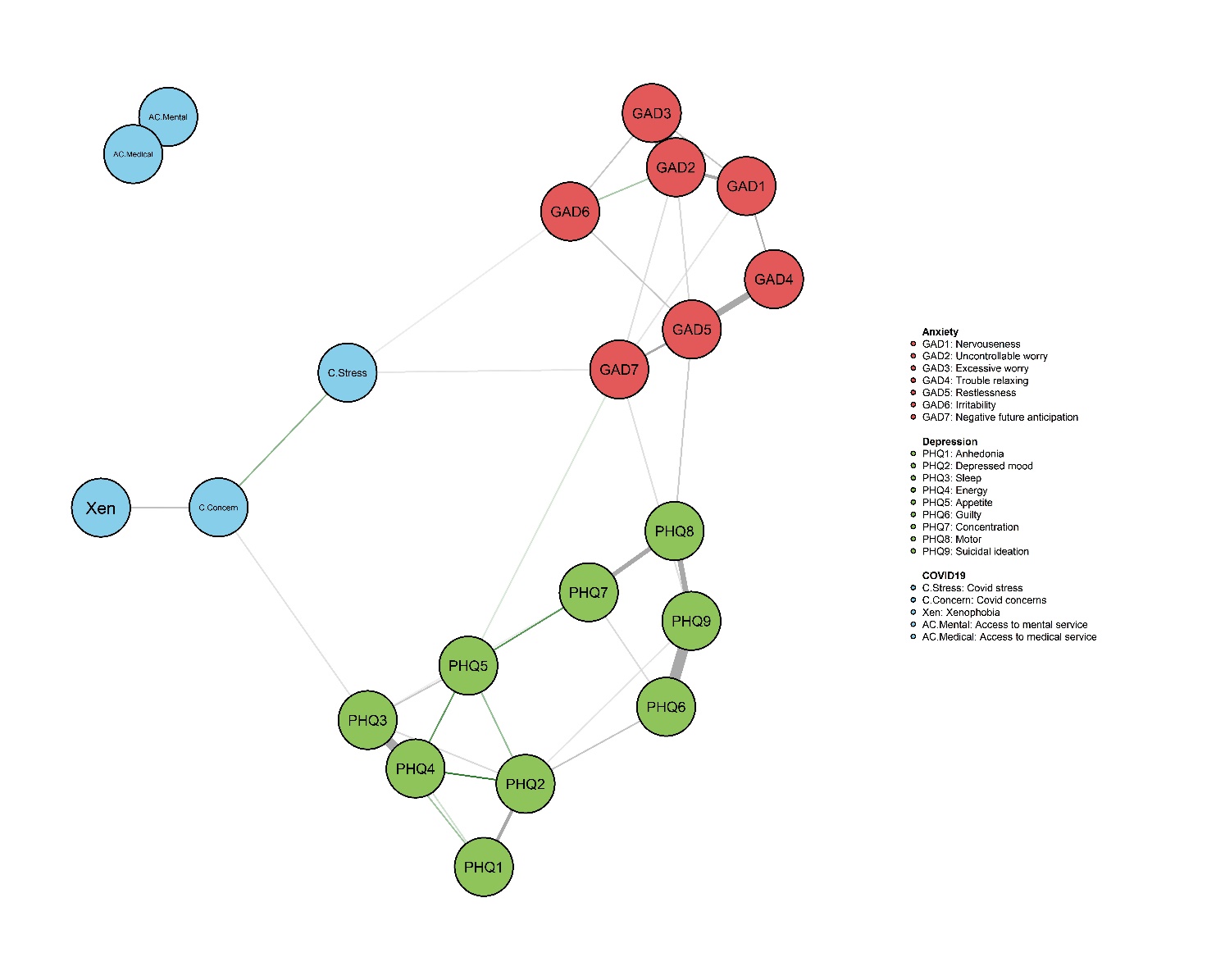


**Fig. S7.** Estimated network of South Korea after controlling for age and gender.


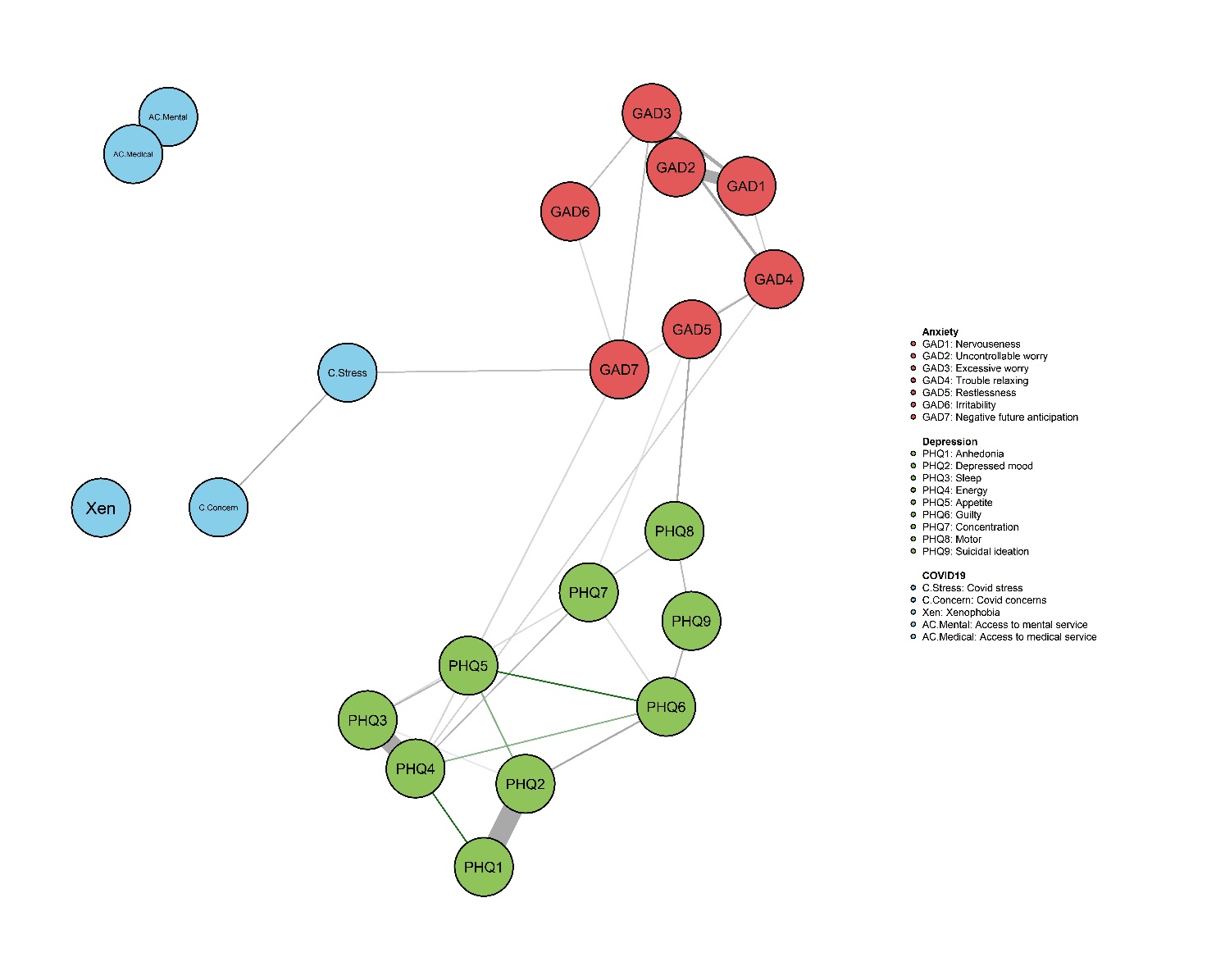


**Fig. S8.** Estimated network of the United States after controlling for age and gender.
